# Photobiont Relationships and Phylogenetic History of *Dermatocarpon luridum* var. *luridum* and Related *Dermatocarpon* Species

**DOI:** 10.3390/plants1020039

**Published:** 2012-10-10

**Authors:** Kyle M. Fontaine, Andreas Beck, Elfie Stocker-Wörgötter, Michele D. Piercey-Normore

**Affiliations:** 1 Department of Biological Sciences, University of Manitoba, Winnipeg, Manitoba, R3T 2N2, Canada; E-Mail: umfontak@cc.umanitoba.ca; 2 Botanische Staatssammlung München, Menzinger Strasse 67, D-80638 München, Germany; E-Mail: beck@bsm.mwn.de; 3 Department of Organismic Biology, Ecology and Diversity of Plants, University of Salzburg, Hellbrunner Strasse 34, A-5020 Salzburg, Austria; E-Mail: Elfriede.Stocker@sbg.ac.at

**Keywords:** sub-aquatic lichen, intercontinental, nuclear ribosomal DNA, phylogeny, algal switching, long distance dispersal

## Abstract

Members of the genus *Dermatocarpon* are widespread throughout the Northern Hemisphere along the edge of lakes, rivers and streams, and are subject to abiotic conditions reflecting both aquatic and terrestrial environments. Little is known about the evolutionary relationships within the genus and between continents. Investigation of the photobiont(s) associated with sub-aquatic and terrestrial *Dermatocarpon* species may reveal habitat requirements of the photobiont and the ability for fungal species to share the same photobiont species under different habitat conditions. The focus of our study was todetermine the relationship between Canadian and Austrian *Dermatocarpon luridum* var. *luridum* along with three additional sub-aquatic *Dermatocarpon* species, and to determine the species of photobionts that associate with *D. luridum* var. *luridum*. Culture experiments were performed to identify the photobionts. In addition, the question of the algal sharing potential regarding different species of *Dermatocarpon* was addressed. Specimens were collected from four lakes in northwestern Manitoba, Canada and three streams in Austria. Three Canadian and four Austrian thalli of *D. luridum* var. *luridum* were selected for algal culturing. The nuclear Internal Transcribed Spacer (ITS) rDNA gene of the fungal partner along with the algal ITS rDNA gene was sequenced to confirm the identity of the lichen/photobiont and afterwards the same data sets were used in phylogenetic analyses to assess algal sharing. The green algal photobiont was identified as *Diplosphaera chodatii* (Trebouxiophyceae). The phylogenetic analyses of Canadian and Austrian *D. luridum* var. *luridum* revealed that ITS sequences are identical despite the vast geographic distance. Phylogenetic placement of *D. luridum* var. *decipiens* and *D. arnoldianum* suggested that a re-examination of the species status might be necessary. This study concluded that additional photobiont culture experiments should be conducted to answer the question of whether multiple photobionts are present within the genus *Dermatocarpon*.

## 1. Introduction

The vast majority of lichens involve the association of a heterotrophic fungus of the Ascomycota and a photoautotrophic alga of the Chlorophyta [1]. Terrestrial ecosystems harbor nearly all known lichens, while some occupy fully or partially submerged rocks of marine or freshwater lake, river/stream and spring ecosystems [2,3]. Considerably less knowledge exists about aquatic and sub-aquatic lichens than aquatic fungi and terrestrial lichens. The majority of lichen species that live in or associated with aquatic ecosystems are systematically positioned within the Verrucariaceae. Many genera within the Verrucariaceae do not form monophyletic clades, but the genus *Dermatocarpon* Eschw. is considered to be monophyletic [4,5] with an umbilicate growth habit, thick-walled lower cortical cells of the thallus, pycnidia, immersed thalloid perithecia containing eight simple spores per ascus, and an absence of hymenial algae in the perithecia as found in species of *Endocarpon* and *Staurothele* [5]. While species of *Dermatocarpon* are globally distributed, North America has 19 to 24 species [6], and Austria has 6 species [7]. Some *Dermatocarpon* species colonize rock substrata that are associated with watercourses, which periodically inundate the lichen thalli; such lichens are considered “sub-aquatic” lichens [8,9]. Other members of *Dermatocarpon* are not associated with watercourses and live on rocks and occasionally on soil. In general, members of this genus exhibit rather diverse morphologies, while other commonly known sub-aquatic species include: *Dermatocarpon arnoldianum*, *D. luridum* var. *luridum* and var. *decipiens*, *D. meiophyllizum* and *D. rivulorum*, that share more or less similar morphological characters. Even sub-aquatic *Dermatocarpon* species such as *D. luridum* are not fully submerged like the aquatic lichens, *Peltigera gowardii* and *P. hydrothyria*. A comprehensive taxonomic treatment of the genus *Dermatocarpon* was published by Amtoft *et al*. [10] and Heiđmarsson [11,12].

Symbiotic interactions between lichenized fungi and their algal partners are described by their degree of selectivity and specificity [13,14]. Selectivity is the preferred interaction between two organisms [15,16] or the frequency of compatible symbiotic partners in lichen associations [17]. Selectivity is assessed from the perspective of one partner in lichen associations and the level of selectivity can be graded as high to low [13]. Specificity is defined as, the coevolutionary pattern between symbionts and it is the result of the selectivity of the partaking bionts [13,16]. Specificity refers to the taxonomic range of acceptable partners, which could be influenced by the environment [17]. Like selectivity, specificity is rank ordered from high to low from the aspect of the fungus and alga, respectively. Some genera of the Verrucariaceae express low selectivity (e.g., *Bagliettoa* sp. and *Verrucaria* sp.), such that they are known to associate with multiple genera of green algae, of different phylogenetically assigned families of photobionts [18]. However, the genus *Verrucaria* is not a good example for selectivity because it is polyphyletic. The model generated by Yahr *et al.* [19] summarized the mycobiont-photobiont relationship (between *Cladonia perforata* and *Asterochloris*) and the intricate systems that structure these interactions.

The diversity of photobiont partners associated with lichen-forming fungi in the Verrucariaceae is thought to be among the greatest of all lichen fungi [20,21]. Recent phylogenetic investigations of photobiont algae using nuclear small subunit and ribulose-bisphosphate carboxylase gene sequences by Thüs *et al.* [18] revealed photobionts in the genera *Stichococcus* and *Diplosphaera* associated with the fungi of the Verrucariaceae. The photobiont of *Dermatocarpon luridum* and several other lichens were phylogenetically identified as belonging to the genus *Diplosphaera* [18]. However, culture experiments of *Dermatocarpon* photobionts were not performed in this study and their identification was based only on nucleotide sequences despite morphological based identifications having been performed by Thüs and Schultz [22]. Early culture experiments in the Verrucariaceae investigated the squamulose rock/concrete inhabiting lichen *Endocarpon pussilum* and crustose rock inhabiting *Staurothele clopima* [23]. Further experiments, performing an “artificial” re-synthesis between the isolated lichen fungus and algae were conducted with *Dermatocarpon miniatum* [24], as well as with *Verrucaria macrostoma* [25,26]. The algal symbionts of *Dermatocarpon miniatum* were identified as *Hyalococcus dermatocarponis*. The photobionts of *Endocarpon pusillum* were already known as *Stichococcus diplosphaera*, whereas the algal partner associated with *Verrucaria macrostoma* was unidentified at this time. In these early investigations, it was found that some algal species, although associated with members of the fungal family Verrucariaceae were incapable of re-synthesizing with other lichen mycobionts. Thus, the natural pairing and interaction of algal symbionts with the “appropriate” mycobionts was thought to be species specific [23]. Certainly, such hypotheses have to be tested *de-novo* with the use of nucleotide sequence markers. But until recently, there is little known about the photobionts associated with fungi of the Verrucariales.

The major objective of this study was to determine the relationship between Canadian and Austrian *Dermatocarpon luridum* var. *luridum* along with three additional sub-aquatic *Dermatocarpon* species. Phylogenetic analysis was expected to shed light on the uncertain taxonomic status of sub-aquatic *Dermatocarpon* species, *D. arnoldianum* and *D. luridum* var *. decipiens*, which until now have eluded molecular investigation. This is the first study to examine the selected sub-aquatic *Dermatocarpon* species and their photobionts simultaneously using a DNA sequence marker as the framework. Further goals were to determine the species of photobiont(s) that associate with *D. luridum* var. *luridum* and finally to investigate the algal sharing potential of selected species of the genus *Dermatocarpon.*

## 2. Results

### 2.1. Microscopy

**Figure 1:**
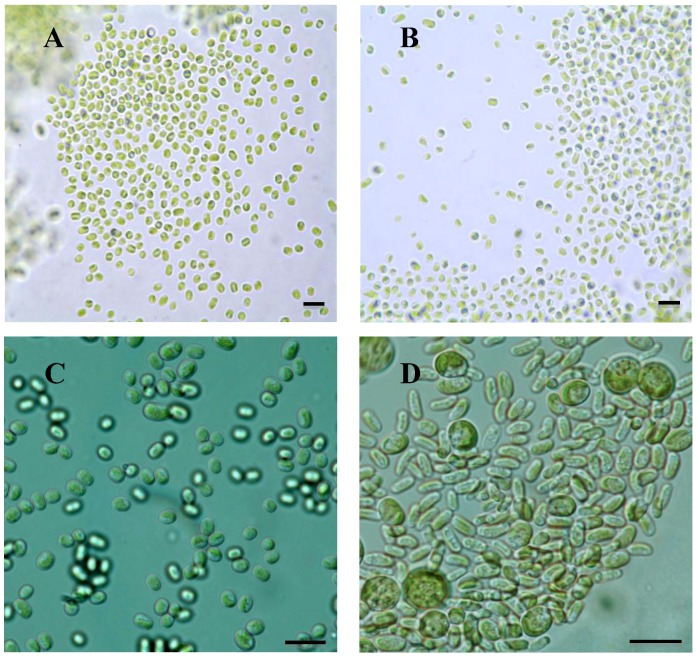
Photobionts isolated from *Dermatocarpon luridum* var. *luridum* and *D. luridum* var. *decipiens* grown in liquid medium. (**A–C**) *Diplosphaera chodatii* isolated from *D. luridum* var. *luridum* (DV4-1, P28-1 and M3-1, respectively); (**D**) Algal cells isolated from *D. luridum* var. *luridum* (Ne18-1). Scale bars = 12 μm.

Algal cells were isolated and cultured from *D. luridum* var. *luridum* showing spherical (4–5 µm) to oval (4–8 µm × 3–5 µm) cell shape with the presence of a parietal plate shaped chloroplast (Figure 1A–C). Algal cultures were obtained from four Austrian collected samples and two Canadian samples (Table 1) that were morphologically identified as *Diplosphaera chodatii* (e.g., Figure 1A–C). An additional algal culture, which was isolated from *D. luridum* var. *luridum* thallus collected in Canada, revealed two different algal cell types (Figure 1D). The first cell type is comprised of spherical to oblate spheroid (7.5–11.5 µm length) shaped cells with the presence of a parietal chloroplast and many globular bodies throughout the cell. The second type is oval to oblong (6–8 µm length) with a width equal to or slightly greater than the width observed in cells of the *D. chodatii* cultures. The second cell type presented characteristics similar to the characteristics described for the first cell type.

### 2.2. Mycobiont Internal Transcribed Spacer (ITS) Phylogeny

**Figure 2:**
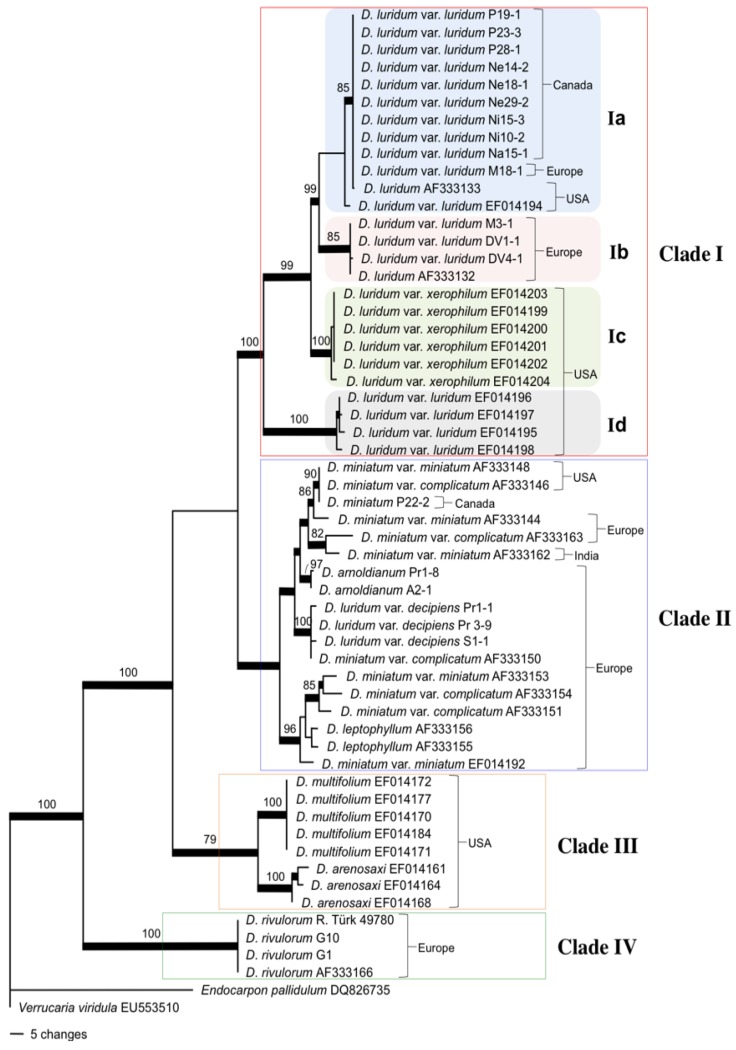
One of 32 most parsimonious phylograms showing the phylogenetic placement of *Dermatocarpon* species from this study relative to species of *Dermatocarpon* from the NCBI GenBank database. The tree is constructed based on the ITS rDNA nucleotide sequence of the mycobiont of sub-aquatic and terrestrial *Dermatocapon* specimens. Numbers above the branch internodes are bootstrap values greater than 70% as presented in PAUP, and thickened branch internodes are supported by ≥95% posterior probability as presented from Bayesian analysis in Mr. Bayes. Taxon identifications are presented followed by collection number or NCBI GenBank Accession numbers. The outgroup was designated as *Endocarpon pallidulum* and *Verrucaria viridula*.

One of 32 most parsimonious trees (Figure 2) summarizes the phylogenetic placement of 22 Internal Transcribed Spacer (ITS) nuclear ribosomal DNA (rDNA) sequences obtained from this study and 36 NCBI GenBank sequences; two of which were used to root the tree. From a dataset of 451 total characters, 140 were parsimony-informative and the final trees shared a tree length of 376 changes with CI and RI scores of 0.6968 and 0.9132, respectively.

The mycobiont tree may be interpreted by identifying four primary clades (Clades I, II, III, and IV), all of which are well supported by bootstrap and posterior probability support. Clade I is supported by 99% bootstrap and 100% posterior probability, and is comprised of all *D. luridum* samples including two varieties. *D. luridum* var. *decipiens* samples are outside this clade and fall into Clade II, which also includes: *Dermatocarpon miniatum* var. *miniatum*, *D. miniatum* var. *complicatum*, *D. arnoldianum*, and *D. leptophyllum*. With the exception of this intraspecific taxon, *D. luridum* might be considered a monophyletic group.

Clade I may be divided into four sub-clades: a, b, c and d. There are 12 samples that constitute sub-clade Ia with 85% bootstrap and 100% posterior probability support. Four samples constitute sub-clade Ib with 85% bootstrap and 99% posterior probability support. Both sub-clades Ia and Ib represent the species *D. luridum* var. *luridum* and are supported with 99% bootstrap and 85% posterior probability support. One sample in sub-clade Ia (M18-1) is from Austria and all others from North America (Manitoba, Minnesota and North Carolina). Sub-clade Ib represents additional *Dermatocapon luridum* var. *luridum* sequences from Europe (Austria and Sweden) and is well supported with 85% bootstrap and 99% posterior probability supports. NCBI GenBank fungal ITS sequences of *Dermatocarpon luridum* var. *luridum* representing individuals of the Southern U.S. comprise I I sub-clade Id. These samples are segregated from the *D. luridum* var. *luridum* collected from Northern Canada and U.S. (sub-clade Ia) and Austria and Sweden (sub-clade Ib). Strong bootstrap and posterior probability, both at 100%, support the placement and segregation of the Southern U.S. from the rest of the *D. luridum* var. *luridum* samples. There are six taxa from NCBI GenBank representing *D. luridum* var. *xerophilum* that comprise sub-clade Ic which is supported by 100% bootstrap and posterior probability support.

Clade II is supported by 95% posterior probability but the taxa: *Dermatocarpon miniatum* var. *miniatum*, *D. miniatum* var. *complicatum*, *D. luridum* var. *decipiens* and *D. leptophyllum* comprising Clade II, with the exception of *D. arnoldianum*,are not monophyletic. Clade III is represented by two monophyletic species *D. multifolium* and *D. arenosaxi* each with 100% support. Clade IV is also monophyletic being comprised of *D. rivulorum* with 100% support.

### 2.3. Photobiont ITS Phylogeny

**Figure 3:**
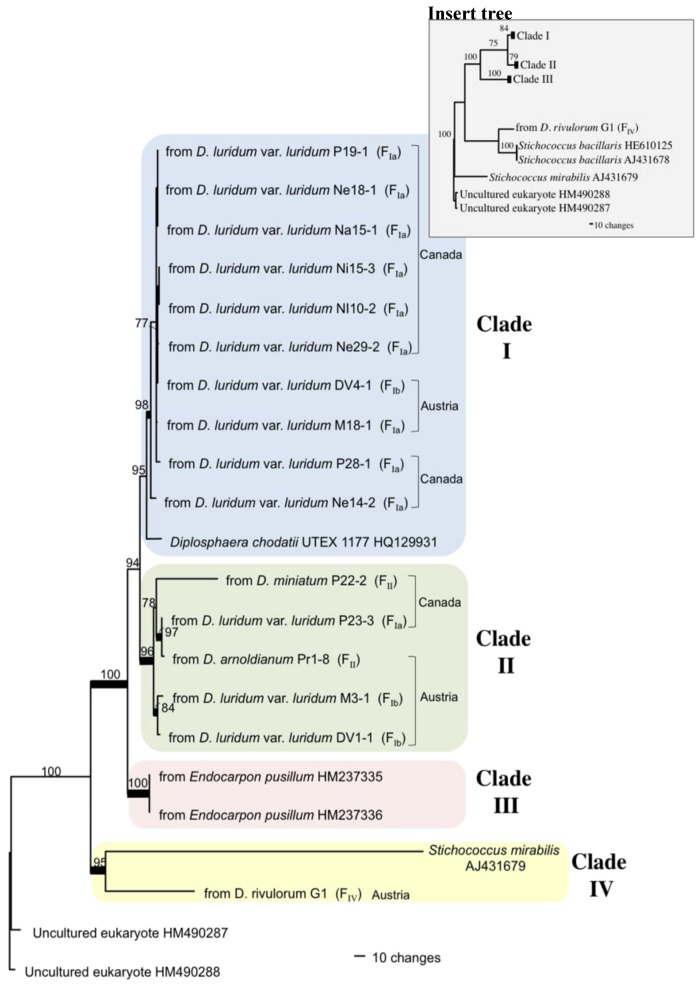
One of nine most parsimonious phylograms showing the phylogenetic placement of the photobiont of the *Dermatocarpon luridum* var. *luridum* and related lichens obtained from this study with sequences of *Diplosphaera chodatii* and similar nucleotide sequences obtained from NCBI GenBank database. The tree is constructed based on the ITS rDNA nucleotide sequences. Numbers above the branch internodes are bootstrap values ≥70%, and thickened branch internodes are supported by ≥95% posterior probability. Samples are presented followed by collection number for those collected in this study or NCBI GenBank Accession numbers for those retrieved from GenBank database. Information presented within parentheses represents the clade from the fungal tree containing the mycobiont from which the photobiont was associated. Two uncultured eukaryotes from NCBI GenBank were chosen as the outgroup.

The ITS rDNA phylogeny represents sequences from 22 algal samples (Figure 3), 16 sequences from this study, and six NCBI GenBank accessions. One of nine most parsimonious trees (Figure 3) is presented from a data set of 699 characters, of which 178 were parsimony-informative characters. The tree length is 707 changes and CI and RI scores were 0.8925 and 0.8281, respectively. Since the Neighbor Joining (NJ) and Maximum Likelihood (ML) trees were consistent with those of the Maximum Parsimony (MP) and Bayesian analyses, the NJ and ML trees are not presented.

Four highly supported clades are presented in the algal phylogenetic tree (Figure 3). Clade I is supported by 95% bootstrap support and it contains algal sequences representing both Austrian and Canadian (Manitoban) photobionts. This clade is composed of the photobionts associated with *D. luridum* var. *luridum* and one NCBI GenBank reference. Clade II, supported by 96% bootstrap support and 95% posterior probability support, contains the algae of three different taxa, *D. miniatum*, *D. luridum* var. *luridum* and *D. arnoldianum*. Algae from three species of *Dermatocarpon*, in Clades I and II form a highly supported group with 94% bootstrap support and cluster with the UTEX sample of *Diplosphaera chodatii*. Clade III contains two NCBI GenBank accessions of *Stichococcus* with 100% of both bootstrap and posterior probability. Clade IV contains a *Stichococcus* species and the alga from *D. rivulorum*, and is supported by 95% bootstrap support and 100% posterior probability support.

Algal clade I are all photobionts of *D. luridum* var. *luridum* from Fungal Clade Ia (Figure 3; F_Ia_), with the exception of one DV4-1 of Fungal Clade Ib (F_Ib_). Algal Clade II contains algae that associate with fungal species that are in three different fungal clades (Figure 3; F_Ia_, F_Ib_, and F_II_).

The insert photo (Figure 3) reveals how the addition of two NCBI GenBank sequences of *Stichococcus bacillaris* disrupts the clade consisting of *Stichococcus mirabilis* and the photobiont sequence from *D. rivulorum.* The photobiont from *D. rivulorum* is grouped in a Clade with the two *S. bacillaris* sequences*.* There is 100% bootstrap support for the pairing of the two *S. bacillaris* sequences. The generation of Clades I, II, and III was identical with 100% bootstrap support. Clade I and II are supported by 84% and 79%, respectively, which are lower support values than the primary tree. Clade III is supported once again by 100% bootstrap support.

## 3. Discussion

### 3.1. Intercontinental Gene Flow

The finding that fungal sequences from Austria clustered with sequences from North American specimens (Figure 2) suggested that gene flow of *D. luridum* var. *luridum* occurred between continents and is likely ongoing (Figure 2 and Figure 3). Other studies of lichen phylogenetic differentiation at the global scale have provided mixed results. The crustose lichen *Porpidia flavicunda* was shown to share identical fungal haplotypes between Northern North America, Greenland and Northern Europe [27] and was hypothesized to have undergone long distance dispersal. Geographic segregation between continents was shown to occur for *Lobaria pulmonaria* [28] and for *Letharia vulpina* [29]. Both local variation within populations and variation between populations especially as distance increased suggested isolation by distance for *Lobaria pulmonaria*. Our study suggests low levels of variation in global and local populations of *Dermatocarpon luridum* var. *luridum* with some sharing between continents, but the small sample size does not support conclusive comments to be made on the extent of gene flow. Since long distance dispersal of lichen diaspores was hypothesized for *Cetraria aculeata* and *Cavernularia hultenii* [27,30,31], this may also be true for the dispersal of the mycobiont, *Dermatocarpon luridum* var. *luridum*. The sharing of genetic information between photobiont populations associated with *D. luridum* var. *luridum* from both continents is implied, but further study is underway to determine the extent of photobiont exchange/movement between continents.

### 3.2. Fungal Evolution: D. luridum *and* D. miniatum Are Paraphyletic

The fungal phylogeny obtained from the ITS sequence data reveals the paraphyletic nature of both *Dermatocarpon luridum* and *Dermatocarpon miniatum* (Figure 2). The placement of *D. luridum* var. *decipiens* into Clade II makes the species paraphyletic. *D. luridum* var. *decipiens* may belong as varietal status within *D. miniatum*. The taxonomic status of *D. arnoldianum* was questioned by Heiđmarsson [11], suggesting it be incorporated into the species as a variety of *Dermarocarpon miniatum* [11]. In his review, Heiđmarsson [11] mentions the prior troubles of taxonomically classifying *D. arnoldianum* by Degelius [32] based on morphological character states and interpretations of iodine reactions relative to *D. linkolae*. The placement of *D. arnoldianum* and *D. luridum* var. *decipiens*, nested within Clade II suggests that the species maybe more appropriately considered subspecies of *D. miniatum* rather than independent species. The use of the ITS rDNA gene to address species delimitation is useful because it contains regions that are faster evolving than the surrounding subunit sequences. The ITS gene is routinely used as a phylogenetic marker for fungal species and genera [33] and it has been proposed for use as a bar coding gene [34]. Limitations of the locus include the use of the ITS rDNA as a single marker where additional markers will provide more data for phylogenetic reconstruction. Also, the potential failure of concerted evolution in the multiple tandem repeating units and paralogous ITS regions may obscure phylogenetic relationships [35]. Further study is also needed to resolve *D. miniatum* var. *complicatum* and var. *miniatum* since all samples in this tree were obtained from NCBI GenBank database.

Although *D. rivulorum* and *D. luridum* are similar in morphology [36], *D. rivulorum* is the most basal of the seven species in the tree (Figure 2). *Dermatocarpon rivulorum* and *D. luridum/D. miniatum* may have evolved from an ancestor with similar sub-aquatic habitat requirements and adapted to a terrestrial environment while maintaining the same photobiont. The aquatic nature of the lichen may have been lost multiple times through evolution of these morphologically similar species. However, the limited sample size in this study prevents an assessment of the ancestral state reconstruction of aquatic and terrestrial species.

### 3.3. Algal Identity

The microscopic analysis of the six cultured photobiont isolates from *Dermatocarpon luridum* var. *luridum* and nucleotide sequencing analyses suggest these algae are *Diplosphaera chodatii* Bialosuknia. The morphology of the cells cultured in this experiment (Figure 1) are consistent with those shown by Zhang and Wei [37], photobiont culture isolates from *Endocarpon pusillum* and the UTEX algal collection 1177 of *D. chodatii*. Algal identity is further supported by the cell morphology and dimension information reported for *Diplosphaera chodatii* (syn. *Stichococcus diplosphaera*) by Ahmadjian and Heikkila [23] in the hymenium of *Endocarpon pusillum.*

Blast search in NCBI GenBank of ITS gene sequences of the photobiont of *D. luridum* var. *luridum* obtained from this study provided six significantly similar matches with query coverage greater than 80%, which have been incorporated into the algal phylogeny (Figure 3). The sequences of *Diplosphaera chodatii* UTEX 1177 and those generated by Zhang and Wei [37] were most similar to our sequences. Three *Stichococcus* sequences with lower similarity were also identified through the BLAST search. The phylogenetic analysis included our algal ITS nrDNA sequences combined with the six NCBI GenBank accessions that supported the morphological identification. The strongly supported clades support that *D. chodatii* is the preferred photobiont of *Dermatocarpon luridum* var. *luridum, Dermatocarpon arnoldianum* and *Dermatocarpon miniatum* [18,38].

One of the cultures obtained from *D. luridum* var. *luridum* (Ne18-1; Figure 1) contained two types of algal cells (autospores and mature vegetative cells). The morphology does not disagree with that of the green alga *Elliptochloris bilobata* Tschermak-Woess (Figure 1) even though the ITS sequence was amplified from *D. chodatii*. *Elliptochloris bilobata* was earlier shown to be a photobiont of *Verrucaria sublobulata* [18] and is comprised of the morphological features shown in Figure 1. However, the algal cell types observed in the culture (Figure 1) were not observed within the lichen thallus. The finding of an additional algal species using culture methods may be explained if *D. luridum* var. *luridum* can associate with more than one photobiont simultaneously; if *Elliptochloris bilobata*, was present on the thallus as a free-living alga. Even though the thallus was examined and washed before culturing, small amounts of an additional species could be selected under certain culture conditions. Other studies have shown multiple photobionts in one culture [39] and multiple photobionts or photobiont genotypes present in the same thallus [16,17,40,41,42]. The phylogeny in Thüs *et al.* [18] shows that *Diplosphaera* sp. is shared with four lichen forming fungi including *D. miniatum*, supporting the findings of this study. The re-synthesis experiments of Stocker-Wörgötter and Türk [24] identified the photobiont associated with *Dermatocarpon miniatum* to be *Hyalococcus dermatocarponis* H. Warén, which does not disagree with the findings in this study. The low selectivity of *D. luridum* and *D. miniatum* provides an explanation for the fungal partner associating with different algal partners in different geographic locations. *Hyalococcus dermatocarponis* was also isolated from *Dermatocarpon luridum* (syn. *Dermatocarpon fluviatilis*) and deposited in the UTEX algal collection (UTEX number 908) by V. Ahmadjian. The finding that six of the seven cultures were morphologically identified as *D. chodatii* provides support for a one-fungus and one-photobiont hypothesis, but it does not eliminate the possibility that more than one photobiont may associate with *D. luridum*.

### 3.4. Photobiont Evolution and Algal Selection

The broad geographic origin of the algal sequences from *Dermatocarpon* species that fall within Clades I and II (Figure 3) suggests that there may be multiple strains of the alga distributed throughout North America and Europe. This broad distribution may be explained by the low selectivity of *D. chodatii* by three mycobiont species, and that in sexual reproduction the fungal ascospores must come into contact with a different compatible algal strain at each dispersal event. Lichen asexual propagules such as isidia and soredia have been hypothesized to serve as vectors for photobiont dispersal [16,40,43] to promote algal dispersal and algal switching. Members of *Verrucaria*, *Staurothele* and *Endocarpon* illustrate another means of algal dispersal by releasing the photobiont into the environment together with ascospores [18]. As with all sexually reproducing lichens, ascospores must come in contact with a suitable alga in order to establish a symbiosis. The ability to tolerate lengths of desiccation stress up to and exceeding two months [37] and their free-living presence in soils, on bark, on rock and as epiphytes on lichens and other living and dead plants [44,45,46,47,48,49] increases the likelihood that spores will come in contact with *D. chodatii* for a symbiosis to establish.

Fungal and algal taxa from both Canada and Austria are placed throughout the trees, suggesting that fungi from both continents share some of the same photobiont species. Additionally, the clustering of algal sequences obtained from different *Dermatocarpon* species suggests sharing of algal genotypes among *Dermatocarpon* taxa and low specificity. *Dermatocarpon miniatum*, a terrestrial species, shares the same photobiont, *Diplosphaera chodatii*, as *Dermatocarpon luridum* var. *luridum* and *Dermatocarpon arnoldianum*, two sub-aquatic species [18,38]. The sub-aquatic *Dermatocarpon luridum* var. *luridum* and terrestrial *Dermatocarpon miniatum* are typically separated by physical distance from one another, where *D. luridum* is located in the submerged zone and *D. miniatum* is in the upper splash zone [50] addressing our original question as to whether they share the same alga in these different habitats.

Both algal and fungal phylogenies place *Dermatocarpon rivulorum* and its algal partner in a basal position to other taxa (Figure 2). Since the DNA sequences of the algal and fungal partners were obtained from the same lichen thallus extract, it is possible that the sequence in the algal tree may represent an epiphyte or contaminant by a *Stichococcoid-*like alga. Assuming that the photobiont sequence is from *D. rivulorum*, the placement of the photobiont ITS sequence from *Dermatocarpon rivulorum* in the basal position in the tree with *Stichococcus* spp. suggests that the alga that associates with *D. rivulorum* may be a *Stichococcoid-*like alga. The degree of morphological similarity between *Stichococcus* species and *Diploshpaera* species greatly exceeds the dissimilarity. One difference is the formation of two-celled or short chain clusters [51]. However, the absence of morphological evidence and the long-branch connecting *Stichococcus* (Figure 3) might suggest that the photobiont associated with *D. rivulorum* could be a *Diplosphaera-*like alga. Long-branch attraction [52] might also account for the placement of *Stichococcus* and photobiont of *D. rivulorum* together in the tree. Culture evidence and additional molecular analysis of cultured photobionts and thallus extracted DNA may help to resolve this issue. The photobiont genus associated with *Dermatocarpon rivulorum* was not reported by Thüs *et al.* [18]; however, Nascimbene *et al.* [3] stated the photobiont is *Diplosphaera chodatii* [38]. Thüs and Schultz [22] also indicated that the photobiont of *D. rivulorum* is *D. chodatii*. The divergence of the photobiont associated with *D. rivulorum* in the algal tree suggests that the species of photobiont is more specific to *D. rivulorum* than to *D. luridum* or *D. miniatum* and may reflect the limit of algal sharing between fungal taxa. Since both *D. rivulorum* and *D. luridum* var. *luridum* grow in similar habitats and in close proximity to one another, it is reasonable to assume that the two fungi associate with the same photobiont species. Even though the single sequence in this study suggests a distant relationship between photobionts of *D. luridum* and *D. rivulorum*, the identity of the *D. rivulorum* photobiont(s) needs more investigation.

## 4. Experimental Section

### 4.1. Lichen Material

Four lakes in west central Manitoba, Canada, and five locations in Austria were sampled for *D. luridum* var. *luridum* and other *Dermatocarpon* lichens. In Canada, ten specimens were collected (Table 1). Thirteen samples were collected from five locations in Austria (Table 1). All lichen thalli were moistened to aid the scraping of the thalli off rock surfaces and reduce damage to the thalli before they were air dried and processed. All vouchers (Table 1) are maintained in the cryptogamic division of the University of Manitoba herbarium (WIN), Winnipeg, Manitoba, Canada.

**Table 1:** List of samples and sequence sources for symbionts used in this study, including collection information and accession (Acc.) numbers for the DNA sequences deposited to NCBI GenBank. NS is not sequenced.

Taxon identification	Sample collection number or source	Collection site	Fungal ITS Acc.	Algal ITS Acc.
*Dermatocarpon arenosaxi*	Amtoft *et al.* [10]	USA, Missouri	EF014161	NS
*Dermatocarpon arenosaxi*	Amtoft *et al.* [10]	USA, Missouri	EF014164	NS
*Dermatocarpon arenosaxi*	Amtoft *et al.* [10]	USA, Arkansas	EF014168	NS
*Dermatocarpon arnoldianum*	Fontaine A2-1	Austria, Salzburg, Preberkessel, 2011; 47°12'47"N; 13°51'10"E	JX645038	NS
*Dermatocarpon arnoldianum*	Fontaine Pr1-8	Austria, Salzburg, Preberkessel, 2011; 47°12'37''N; 13°51'05''W	JX645037	JX645019
*Dermatocarpon leptophyllum*	Heiđmarsson [12]	Sweden	AF333155	NS
*Dermatocarpon leptophyllum*	Heiđmarsson [12]	Sweden	AF333156	NS
*Dermatocarpon luridum*	Heiđmarsson [12]	USA, Minnesota	AF333133	NS
*Dermatocarpon luridum*	Heiđmarsson [12]	Sweden	AF333132	NS
*Dermatocarpon luridum* var. *decipiens* *	Fontaine Pr1-1	Austria, Salzburg, Preberkessel, 2011; 47°12'37''N; 13°51'05''W	JX645039	NS
*Dermatocarpon luridum* var. *decipiens*	Fontaine Pr3-9	Austria, Salzburg, Preberkessel, 2011; 47°13'16"N; 13°51'13"E	JX645040	NS
*Dermatocarpon luridum* var. *decipiens*	Fontaine S1-1	Austria, Styria, Schladminger Tauern, 2011; 47°16'17''N; 13°43'47"E	JX645041	NS
*Dermatocarpon luridum* var. *luriudm*	Amtoft *et al.* [10]	USA, North Carolina	EF014194	NS
*Dermatocarpon luridum* var. *luridum*	Amtoft *et al.* [10]	USA, Arkansas	EF014195	NS
*Dermatocarpon luridum* var. *luridum*	Amtoft *et al.* [10]	USA, Missouri	EF014196	NS
*Dermatocarpon luridum* var. *luridum*	Amtoft *et al.* [10]	USA, Alabama	EF014197	NS
*Dermatocarpon luridum* var. *luridum*	Amtoft *et al.* [10]	USA, Missouri	EF014198	NS
*Dermatocarpon luridum* var. *luridum*	Fontaine P19-1	Canada, Manitoba, Payuk Lake, 2010; 54°38'31''N; 101°31'40''W	JX645023	JX645008
*Dermatocarpon luridum* var. *luridum*	Fontaine P23-3	Canada, Manitoba, Payuk Lake, 2010; 54°38'31''N; 101° 31'40''W	JX645024	JX645018
*Dermatocarpon luridum* var. *luridum* *	Fontaine P28-1	Canada, Manitoba, Payuk Lake, 2010; 54°38'31''N; 101°31'40''W	JX645025	JX645015
*Dermatocarpon luridum* var. *luridum*	Fontaine Ne14-2	Canada, Manitoba, Neso Lake, 2010; 54°39'51''N; 101°32'44''W	JX645026	JX645016
*Dermatocarpon luridum* var. luridum*	Fontaine Ne18-1	Canada, Manitoba, Neso Lake, 2010; 54°39'51''N; 101°32'44''W	JX645027	JX645009
*Dermatocarpon luridum* var. *luridum*	Fontaine Ne29-2	Canada, Manitoba, Neso Lake, 2010; 54°39'51''N; 101°32'44''W	JX645028	JX645013
*Dermatocarpon luridum* var. *luridum* *	Fontaine Ni15-3	Canada, Manitoba, Nisto Lake, 2010; 54°42'02''N; 101°30'17''W	JX645029	JX645011
*Dermatocarpon luridum* var. *luridum*	Fontaine Ni10-2	Canada, Manitoba, Nisto Lake, 2010; 54°42'02''N; 101°30'17''W	JX645030	JX645012
*Dermatocarpon luridum* var. *luridum*	Fontaine Na15-1	Canada, Manitoba, Naosap Lake, 2010; 54°50'38''N; 101°26'12''W	JX645031	JX645010
*Dermatocarpon luridum* var. *luridum* *	Fontaine M3-1	Austria, Waldaist, 2011; 48°19'44''N; 13°52'28''E	JX645033	JX645020
*Dermatocarpon luridum* var. *luridum* *	Fontaine M18-1	Austria, Waldaist, 2011; 48°23'49''N; 13°35'93''E	JX645032	JX645045
*Dermatocarpon luridum* var. *luridum*	Fontaine DV1-1	Austria, Schlogener Schlinge, 2011; 48°25'59''N; 13°52'28''E	JX645034	JX645021
*Dermatocarpon luridum* var. *luridum* *	Fontaine DV4-1	Austria, Schlogener Schlinge, 2011; 48°25'59''N; 13°52'28''E	JX645035	JX645014
*Dermatocarpon luridum* var. *xerophilum*	Amtoft *et al.* [10]	USA, Missouri	EF014199	NS
*Dermatocarpon luridum* var. *xerophilum*	Amtoft *et al.* [10]	USA, Oklahoma	EF014200	NS
*Dermatocarpon luridum* var. *xerophilum*	Amtoft *et al.* [10]	USA, Arkansas	EF014201	NS
*Dermatocarpon luridum* var. *xerophilum*	Amtoft *et al.* [10]	USA, Arkansas	EF014202	NS
*Dermatocarpon luridum* var. *xerophilum*	Amtoft *et al.* [10]	USA, Arkansas	EF014203	NS
*Dermatocarpon luridum* var. *xerophilum*	Amtoft *et al.* [10]	USA, Arkansas	EF014204	NS
*Dermatocarpon miniatum*	Fontaine P22-2	Canada, Manitoba, Payuk Lake, 2010; 54°38'31''N; 101°31'40''W	JX645036	JX645017
*Dermatocarpon miniatum* var. *complicatum*	Heiđmarsson [12]	USA, Minnesota	AF333146	NS
*Dermatocarpon miniatum* var. *complicatum*	Heiđmarsson [12]	Sweden	AF333150	NS
*Dermatocarpon miniatum* var. *complicatum*	Heiđmarsson [12]	Iceland	AF333151	NS
*Dermatocarpon miniatum* var. *complicatum*	Heiđmarsson [12]	Austria	AF333154	NS
*Dermatocarpon miniatum* var. *complicatum*	Heiđmarsson [12]	Norway	AF333163	NS
*Dermatocarpon miniatum* var. *miniatum*	Heiđmarsson [12]	Sweden	AF333144	NS
*Dermatocarpon miniatum* var. *miniatum*	Heiđmarsson [12]	USA, Minnesota	AF333148	NS
*Dermatocarpon miniatum* var. *miniatum*	Heiđmarsson [12]	Iceland	AF333153	NS
*Dermatocarpon miniatum* var. *miniatum*	Heiđmarsson [12]	India	AF333162	NS
*Dermatocarpon miniatum* var. *miniatum*	Amtoft *et al.* [10]	Wales	EF014192	NS
*Dermatocarpon multifolium*	Amtoft *et al.* [10]	USA, Missouri	EF014170	NS
*Dermatocarpon multifolium*	Amtoft *et al.* [10]	USA, Missouri	EF014171	NS
*Dermatocarpon multifolium*	Amtoft *et al.* [10]	USA, Missouri	EF014172	NS
*Dermatocarpon multifolium*	Amtoft *et al.* [10]	USA, Virginia	EF014177	NS
*Dermatocarpon multifolium*	Amtoft *et al.* [10]	USA, North Carolina	EF014184	NS
*Dermatocarpon rivulorum*	Heiđmarsson [12]	Sweden	AF333166	NS
*Dermatocarpon rivulorum*	Türk 49780	Austria, Carinthia, Hohe Tauern, 2011; 46°59'18''N; 13°15'28''E	JX645042	NS
*Dermatocarpon rivulorum*	Fontaine G1	Austria, Carinthia, Hohe Tauern, 2011; 46°56'13''N; 13°00'25''E	JX645044	JX645022
*Dermatocarpon rivulorum*	Fontaine G10	Austria, Carinthia, Hohe Tauern, 2010; 46°56'13''N; 13°00'25''E	JX645043	NS
*Diplosphaera chodatii*	Zhang and Wei [37]	China	NS	HM237335
*Diplosphaera chodatii*	Zhang and Wei [37]	China	NS	HM237336
*Diplosphaera chodatii*	Zhang and Wei [37]	UTEX culture collection #1177	NS	HQ129931
*Stichococcus bacillaris*	Marin [53]	Unknown	NS	HE610125
*Stichococcus bacillaris*	Unpublished	Unknown	NS	AJ431678
*Stichococcus mirabilis*	Unpublished	Unknown	NS	AJ431679
Uncultured eukaryote	Khan *et al.* [54]	Antarctica	NS	HM490287
Uncultured eukaryote	Khan *et al.* [54]	Antarctica	NS	HM490288

* Specimens used for algal culture experiments.

### 4.2. Photobiont Isolation for Culturing

Photobiont isolation from *Dermatocarpon luridum* var. *luridum* was performed using seven thalli (Table 1) according to the Yamamoto method [55], and isolations were performed using the procedure as described in Stocker-Wörgötter [56] and Stocker-Wörgötter and Hager [57]. A lobe of lichen thallus, approximately 20 mm^2^ was cut into smaller fragments and washed in a 50 mL Erlenmeyer flask containing 25 mL of sterile double distilled water for 15 min on a stir table. One drop of Tween 80 was added to the water and the fragments were washed for an additional 15 minutes on a magnetic stirrer. The lichen thallus was transferred using sterile forceps to a clean 50 mL Erlenmeyer flask and washed in 25 mL of fresh sterile double distilled water for 20 min to remove the Tween 80. 

Under sterile conditions, the washed lichen fragments were then ground in 3–4 mL of sterile water using mortar and pestle which was filtered through a sieve with mesh pore size of 500 µm and the fragments that passed through were then filtered for the second time through a mesh with pore size 150 µm. The filtrate consisting of fragments of lichen “tissue” (about 150 µm) were selected under a stereomicroscope using sterile bamboo sticks and transferred to slanted agar media. 

Woods Hole MBL [58] was used to culture and isolate axenic colonies of the photobiont. Cultures were grown for 2–3 months at 22 °C under a changing light:dark regime of 14:10 h with a light intensity of 100 µE m^−2^s^−1^. Sub-culturing of the photobiont to liquid medium was performed, once the algal colony size reached 2–3 mm diameter. An inoculation needle was flamed and the colony was fractioned into tiny drops. A small number of algae were transferred to a sterile 50 mL Erlenmeyer flask containing 25–30 mL of liquid medium. Single alga isolates were not feasible, because of the small size of the *Diplopshaera* cells. 

### 4.3. Microscopic Examination

To investigate the morphological features of the algal cells in culture, cells were streaked into water on a microscope slide and observed using a Univar Light Microscope (Reichert INC., Vienna, Austria) and photographs were taken with a Canon PowerShot G5 camera (Canon INC., Tokyo, Japan). Algal species cultures were sent to A. Beck and temporarily maintained at the Culture Collection of the Botanische Staatssammlung Munich, Germany.

### 4.4. DNA Extraction, Amplification, and Purification

Extraction of DNA was accomplished using a protocol modified from Grube *et al.* [59]. The ITS rDNA of both the mycobiont and photobiont of 22 *Dermatocarpon* thalli was amplified by Polymerase Chain Reaction (PCR). The DNA was initially amplified using 20 μL reactions to determine the optimal DNA concentration for PCR and then amplified in larger quantities (four to eight 20 μL PCR reactions) for sequencing. One 20 μL reaction consisted of 5–25 ng of genomic DNA, 1× PCR buffer (50 mM KCl; 100 mM Tris-HCl [pH 8.3]), 2.0 mM MgCl_2_, 200 μM of each dNTP, 0.5 μM of each primer, and 2 units of Taq DNA Polymerase (Invitrogen, Burlington, ON, Canada).

The fungal and algal ITS genes were amplified using the fungal specific forward primer ITS1F-5' (5'-CTTGGTCATTTAGAGGAAGTAA-3') [60] and the newly designed algal specific primer STICHO-ITS-F-5' (5'-GGATCATTGAATCTATCAACAAC-3'), respectively, both together with the universal reverse primer ITS4-3' (5'-TCCTCCGCTTATTGATATGC-3') [61]. All amplifications of the ITS genes were performed using a Biometra T-Gradient thermal cycler (Fisher Sci, Nepean, ON, Canada). The PCR cycle used to amplify the fungal ITS consisted of an initial denaturation of the DNA at 94 °C for 3 min; then 30 cycles of denaturation at 94 °C for 1 min, annealing at 61.5 °C for 30 s and extension at 72 °C for 45 s; followed by 6 °C soak. Amplification of the algal ITS was accomplished with an initial denaturation of the DNA at 94 °C for 3 min; then 30 cycles of denaturation at 94 °C for 1 min, annealing at 58.5 °C for 35 s, and extension at 72 °C for 45 s; followed by 6 °C soak.

Precipitation of the combined PCR products was carried out by adding 0.2 volumes of 5 M NaCl and 2.5 volumes of 100% ethanol. The tubes were gently inverted several times, placed in the fridge (4 °C) for 30 min, and centrifuged for 10 min at 13,000 rpm. The supernatant was poured off and the pellet was washed with 300 μL of cold 80% ethanol and the pellets were left to air dry for 30 min. This pellet was dissolved in 20 μL of sterile distilled water and the entire 20 μL PCR volume was electrophoresed on a 1% agarose gel, the bands were excised from the gel, and purified using Promega’s Wizard^®^ SV Gel and PCR Clean-Up System (Promega Corporation, Madison, WI, USA). The purified DNA was resuspended in 35 μL of sterile distilled water, and quantified by gel electrophoresis. Band intensities were compared with the 1650 base pair band (80 ng/μL) of a 1 kb plus DNA ladder (Invitrogen, Burlington, ON, Canada) and visualized using an AlphaImager 2200 transilluminator (Alpha Innotech, Fisher Scientific, Nepean, ON, Canada).

### 4.5. DNA Sequencing

PCR products were cycle sequenced with BigDye v.3.1 (Applied Biosystems, Foster City, CA, USA) following the manufacturer’s directions. Precipitation and cleanup of the cycle sequenced product to remove excess fluorescent dyes was carried out by the EDTA method following manufacturer’s directions, resuspended in Hi-Di formamide (Applied Biosystems, Foster City, CA, USA) and loaded to a 96-well plate for sequencing with an Applied Biosystems 3130 Genetic Analyzer (Applied Biosystems, Foster City, CA, USA).

### 4.6. Data Analysis

Nucleotide sequences obtained from samples collected for this study were edited using Sequencher v. 4.8 (Gene Codes Corporation, Ann Arbor, MI, USA). Sequences retrieved from NCBI GenBank were selected by first performing a nucleotide BLAST [62] search on the entire nucleotide collection using each algal sequence of the photobionts from *D. luridum* var. *luridum* as the query sequence. Only those with significant e-values of 10e^−50^ [63] and high query coverage (>75%) were selected for phylogenetic analysis. Sequences were aligned manually using Se-Al v. 1.0 [64] and two ambiguous regions were delimited based on visual inspection where substitutions were too numerous for a homologous alignment. The alignment was subjected to parsimony analysis in PAUP* 4.0b10 [65] and Bayesian analysis in MrBayes v3.1.2 [66,67]. Four MP analyses were performed for the algal alignment to assess the effect of the ambiguous regions on the phylogeny: (1) one ambiguous region was removed from the analysis; (2) the other ambiguous region was removed from the analysis; (3) both ambiguous regions were removed from the analysis; and (4) both ambiguous regions were included in the analysis. Since the topology was the same and the bootstrap values fluctuated between 3 and 5%, the final analysis presented in the paper includes both ambiguous regions. The fungal data set that was analyzed consisted of 58 fungal ITS sequences, 22 derived from the current study and 36 from NCBI GenBank (Table 1). Likewise, the algal data set that was analyzed consisted of 22 algal ITS sequences, 16 derived from the current study and 6 from NCBI GenBank (Table 1). The fungal outgroup species were *Endocarpon pallidulum* and *Verrucaria viridula*, while the algal outgroup was designated as uncultured Eukaryotic algae. The sequences obtained from this study have been deposited in NCBI GenBank and accession numbers are indicated in Table 1.

Maximum Parsimony settings were set to run a full heuristic search with a tree bisection and reconnection (TBR) branch swapping algorithm, with a stepwise addition and 5,000 replicates (Fungal data set) or 10,000 replicates (Algal data set) with random addition of taxa. Swapping was performed on only the best trees, and only the best trees were kept with the saving of Multrees option off. The most parsimonious trees were analyzed for bootstrap support within PAUP with the following MP settings: 1,000 bootstrap replicates [68] were performed and only scores greater than 70% were presented in the phylogenetic trees. All other settings were the same as the initial heuristic search; all but the random addition of taxa setting, which was set to 10 for the fungal analysis and 100 for the algal analysis. Sequence alignment contained ambiguous regions in the algal data set from base pair positions 76–107 and 415–465 of the unaligned UTEX 1177 (HQ129931) sequence. Each of theses regions were individually excluded from subsequent analyses and then all together excluded to see if tree topology was altered significantly. All ambiguous regions had minimal effect on the topology and clade scores, therefore all character data was used for the phylogenetic analysis presented.

Bayesian analysis was performed following the best evolutionary model estimated with JModeltest 0.1.1 [69,70,71]. Both ITS data sets were analyzed according to a TrN+G (Tamura-Nei plus Gamma model), which was presented as the best model according to hLRTs (Hierarchial Likelihood Ratio Tests) output within JModeltest. This model follows a six parameter with a gamma shaped distribution. The TrN+G analysis parameters were set as follows for the fungal dataset: Lset Base = (0.2398, 0.2504, 0.3044); Nst = 6; Rmat = (1.0000, 4.7694, 1.0000, 1.0000, 1.7829); Rates = Gamma; Shape = 0.4751; and Pinvar = 0. Likewise, the TrN+G analysis parameters were set as follows for the algal dataset: Lset Base = (0.2323, 0.3015, 0.2792); Nst = 6; Rmat = (1.0000, 1.8102, 1.0000, 1.0000, 3.8085); Rates = Gamma; Shape = 0.3942; and Pinvar = 0. The model information for both analyses was incorporated into PAUP for ML Analysis (outcomes not presented). The settings of this model correspond to the GTR+G evolutionary model. Bayesian analyses were performed using 2,000,000 generations and were terminated well after the standard deviation of split frequencies fell below 0.01. The number of trees discarded following the analysis included 500 burn-in trees. Posterior probability scores greater than 90 are reported on the phylogenies. 

A phenetic analysis of both data sets using NJ was also performed in PAUP using the default settings and a Kimura 2-parameter model (outcomes not shown). Outcomes of the ML and Neighbour Joining analyses are not presented because the results were largely congruent with the MP and Bayesian analyses.

## 5. Conclusions

In conclusion, the sub-aquatic nature of the lichen is not indicative of the phylogenetic placement using ITS sequence data. *Dermatocarpon luridum* var. *luridum* specimens collected in Canada and Austria are phylogenetically similar and form two strongly supported clades. Additional specimens from Europe are closely associated with the Austrian representatives, while the additional specimens from North America fall out in three sub-clades. *Dermatocarpon luridum* var. *decipiens* and *Dermatocarpon arnoldianum* are phylogenetically positioned with *Dermatocarpon miniatum*. Taxonomic revision of *D. miniatum* is needed and it is recommended that *D. luridum* var. *decipiens* be investigated as a variety of *D. miniatum*. However, phylogenetic study of all species within the genus is needed before taxonomic revisions should be considered. The use of ITS data alone for reconstruction of a fungal phylogeny may pose problems if the ITS region contains paralogues. The presence of paralogous rDNA regions may overestimate diversity and not truly represent the phylogenetic placement of taxa. The use of further independent markers would be required before proposing taxonomic changes based on an ITS phylogeny. The final sub-aquatic species investigated, *D. rivulorum* is segregated from the rest of the *Dermatocarpon* species as a strongly supported monophyletic clade. Investigation of the algae suggests that *Diplosphaera chodatii* is the photobiont associated with *Dermatocarpon luridum* var. *luridum*, as well as the other species of *Dermatocarpon*. *Dermatocarpon rivulorum*, however is not thought to associate with *Diplospheara chodatii* or at least not one of the strains shown to associate with other *Dermatocarpon* species. The shared ITS sequence similarities of the photobiont between fungal species suggests that algal sharing is occurring within and between fungal species. The presence of common genotypes of both the mycobiont and photobiont in North America and Europe may support long distance dispersal and photobiont replacement.
